# Structural Changes in Thin Keratoconic Corneas Following Crosslinking with Hypotonic Riboflavin: Findings on *In Vivo* Confocal Microscopy

**DOI:** 10.18502/jovr.v16i3.9429

**Published:** 2021-07-29

**Authors:** Aalia Rasool Sufi, M. Soundaram, Nilam Gohil, Jeremy D. Keenan, N. Venkatesh Prajna

**Affiliations:** ^1^Department of Cornea and Refractive Surgery, Aravind Eye Hospital, Madurai, India; ^2^Francis I. Proctor Foundation, University of California, San Francisco; ^3^Department of Ophthalmology, University of California, San Francisco, California, USA

**Keywords:** Collagen Crosslinking, Hypotonic Riboflavin, In Vivo Confocal Microscopy, Keratoconus

## Abstract

**Purpose:**

To report structural changes observable in *in vivo* confocal microscopy (IVCM) in keratoconic corneas < 400 μm treated with hypotonic riboflavin and collagen crosslinking (CXL).

**Methods:**

Ten eyes of ten patients with progressive keratoconus and corneal thickness between 350 and 399 μm underwent CXL with hypotonic riboflavin. IVCM was performed preoperatively and at one month, three months, and six months after the procedure.

**Results:**

IVCM analysis one month postoperatively showed complete absence of the subepithelial nerve plexus with gradual regeneration over six months in 8 of the 10 eyes, and poor regeneration in the remaining 2 eyes. The anterior stroma showed extracellular lacunae and hyper-reflective cytoplasm in a honeycomb appearance signifying edema at one month which gradually decreased over six months post CXL. Stromal keratocyte apoptosis was evident in the anterior stroma in all cases and extended to the posterior stroma in four eyes with gradual regeneration evident at three and six months. The specular endothelial count decreased by 8% (*P* = 0.005) post-CXL, but no corneas developed clinical signs of endothelial trauma.

**Conclusion:**

IVCM analysis of thin corneas after hypotonic CXL showed posterior corneal structural changes. Posterior stromal changes were accompanied by a decrease in the endothelial cell count. This case series was a preliminary feasibility study that might necessitate conducting a well-designed controlled study.

##  INTRODUCTION

Keratoconus is a noninflammatory degenerative condition of the cornea, characterized by progressive corneal thinning and steepening.^[1]^ Though the exact etiology is unknown, alterations in the corneal collagen structure^[2,3]^ and degree of collagen crosslinking (CXL) leads to a structurally weakened corneal tissue.^[4]^ The spectrum of disease can range from mild astigmatism to severe ectasia requiring corneal transplantation.

CXL is a popular method for management of keratoconus. It is a simple technique in which riboflavin and ultraviolet A (UVA) light is applied to the eye; this treatment is postulated to increase the biochemical and biomechanical stability of the cornea^[5]^ by stimulating the CXL.^[6,7]^ Numerous studies have shown that CXL is a safe method that delays or halts the progression of corneal ectasia and reduces the demand for keratoplasty.^[8,9,10]^ The major limitation of CXL has been the potential for endothelial trauma when used in thin corneas, usually defined as those < 400 μm.^[11]^ To address this shortcoming, different modifications of the CXL procedure for thin corneas have been studied, including contact lens-assisted CXL (CACXL), CXL with customized epithelial debridement, and the use of hypotonic riboflavin. Hypotonic riboflavin hydrates the stromal corneal layer, which should theoretically prevent penetration of the CXL treatment to the endothelium.^[12]^

The morphological effects of CXL on the cornea can be evaluated by *in vivo* confocal microscopy (IVCM), which is a noninvasive method used to study the living cornea at the cellular level.^[13,14]^ Several studies have demonstrated significant tissue alterations on confocal microscopy following standard CXL for keratoconus, including more compact lamellar interconnections and biosynthesis of new collagen.^[15,16,17]^ These changes have been theorized to cause strengthening of the corneal collagen.^[18]^ Although the results of IVCM study of the structural modifications in corneas thinner than 400 microns following CACXL^[19]^ and the customized epithelial debridement^[20,21]^ have already been reported, the IVCM findings following hypotonic CXL have not yet been described. The aim of our study was to analyze the corneal structural modifications observable on IVCM following treatment with hypotonic riboflavin and subsequent CXL.

##  METHODS

This prospective study was conducted at Aravind Eye Hospital Madurai, India and adhered to the tenets of Helsinki. Ethical approval was obtained from the institutional review board of Aravind Eye Hospital Madurai. A written informed consent was obtained from all patients before inclusion in the study. Patients who were at least 10 years of age and had keratoconus with preoperative thinnest pachymetry between 350 and 399 μm (assessed with Pentacam, Oculus Optikgeräte GmbH, Wetzlar, Germany) and automated endothelial cell density of 2400 cells/mm ${}^{2}$ or higher (assessed with Konan Specular Microscope X NSP-9900, Konan Medical, Inc., Japan) were eligible for inclusion in the study. Corneas were excluded from the study if any of the following factors were present: corneal scar, history of previous anterior segment surgery, active anterior segment disease possibly affecting the epithelial healing, or active allergic ocular disease. Patients who were pregnant, lactating, or had systemic connective tissue disease were also excluded. Only one eye per patient was included in the study; the selection of which eye to include was based on ocular exclusion criteria, or if both eyes were eligible, then the eye with thinner corneal pachymetry was selected.

### Ocular Assessment

All patients were subjected to a comprehensive preoperative ocular examination, which included measurement of uncorrected distance visual acuity (UCVA), best corrected distance visual acuity (BCVA) and the manifest refraction, as well as an anterior segment slit lamp evaluation and intraocular pressure measurement by noncontact tonometry. Topography and optical pachymetry were then performed using the Pentacam, followed by IVCM using the Heidelberg Retinal Tomograph III equipped with Rostock Corneal Module (HRTIII-RCM; Heidelberg Engineering GmbH, Heidelberg, Germany) and automated endothelial cell density estimation with the Konan Specular Microscope. After completing all tests, a dilated fundus evaluation was performed to rule out any posterior segment pathology. All examinations were repeated in the same fashion at one, three, and six months postoperatively.

### Confocal Microscopy

IVCM was performed with the laser scanning HRT III-RCM, an instrument whose transverse and longitudinal resolution is 2 μm and 4 μm, respectively. Local anesthetic (0.5% proparacaine solution, Aurolab, Madurai) was instilled in the eye and patients were asked to look at an external fixation target. The instrument objective was brought into contact with the center of the cornea using a disposable sterile polymethyl-methacrylate (PMMA) cap filled with a high-viscosity coupling agent (Genteal gel; Novartis pharmaceuticals, Australia). Images of the entire anteroposterior extent of the cornea were taken at the optical center of the cornea. Each individual scan provided a cube of 40 stacked 400 × 400 μm scans measuring approximately 80 microns in depth. Two anterior to posterior scans per patient were taken and the best sequence was selected. An area of inferior peripheral cornea outside the treatment zone was scanned in the same manner to serve as a control.

### Qualitative Confocal Image Analysis

Two independent examiners with clinical experience in treating keratoconus and interpreting *in vivo* confocal images (ARS, MS) reviewed all scans from each study subject, masked to the results of the other examiner and to whether or not the eye had vernal keratoconjunctivitis (VKC) at all visits. Each examiner provided a qualitative assessment of the epithelium, sub-basal nerve plexus, stroma, and endothelium. The graders met before starting their analyses to reach common definitions for epithelial metaplasia, microdot globular cells, dendritic cells, hyperreflectivity, keratocytes, and hyperreflective bands, based on previous publications.^[15,16,17][22][23]^ The first three clear images immediately posterior to Bowman's membrane were defined as the anterior corneal stromal images, and the first three clear images immediately anterior to the endothelium were defined as the posterior corneal stromal images for analysis. Blurred or non-tangential images were excluded.

### Quantitative Confocal Image Analysis

All selected images were de-identified and randomized. Quantitative analysis was subsequently performed by the same two aforementioned graders using the manufacturer's software to manually count all keratocyte nuclei that were in focus; cells whose borders were not completely within the image frame were included on the top and right-hand aspects of the frame and excluded on the bottom and left-hand aspects. Keratocyte density was calculated as the number of cells per area scanned (i.e., 160 mm ${}^{2}$ ); the two graders' density measurements were averaged for each of the six scans per eye, and the median of the three anterior stromal scans and the median of the three posterior stromal scans were used for analysis.

### CXL Procedure

After topical anesthesia using proparacaine 0.5% eye drops, a baseline measurement of the corneal thickness was performed using the Pacscan 300P ultrasonic pachymetry device (Sonomed, New York, USA ). This measurement was then repeated after debriding the central 8 mm of the corneal epithelium. A metal guard was placed at the limbal region to protect the stem cells from the UV radiation and also to act as a well for the instilled riboflavin solution. Distilled water was instilled every 2 min for 10 min in order to swell the cornea, which was confirmed by pachymetry. This was followed by instillation of 0.1% hypotonic riboflavin solution every 2 min for 20 min. Corneal thickness measurements were repeated after application of riboflavin at the thinnest point of the cornea to confirm a value > 400 μm. In cases where the thickness was < 400 μm, hypotonic riboflavin was instilled every 20 sec for another 5 min until the thickness increased to ≥ 400 μm. All cases in this report eventually reached a central thickness of ≥ 400 μm. Subsequently, ultraviolet light of 370 nm wavelength was used to irradiate an 8 mm diameter of the central cornea with an irradiance of 3mW/cm ${}^{2}$ (using the UV-X device, UV-X; Company Peschke, Nürnberg, Germany). During the 30 min of irradiation, hypotonic riboflavin solution was instilled to the cornea every 2 min to maintain the necessary concentration of the solution and the required thickness of the cornea. A drop of 0.5% moxifloxacin solution was instilled at the end of the procedure, followed by application of a bandage soft contact lens (BCL) and instructions to apply moxifloxacin 0.5% four times per day. On the third postoperative day, the BCL was removed and topical loteprednol 0.5% four times per day was added on the seventh postoperative day, which was tapered over three weeks.

### Statistical Analysis

The Wilcoxon signed rank test was used to test the difference between baseline and follow-up visits, with a significance level of 0.05 for each analysis. No correction was made for multiple comparisons in this small hypothesis-generating study. All statistical analyses were performed using Stata 14.2 (College Station, TX, USA).

##  RESULTS

The study population included four females and six males, with a median age of 18 years (interquartile range [IQR] 14–20; range, 12–21). Four patients had a previous episode of VKC. The median thinnest corneal pachymetry measurement on Pentacam was 381 μm (IQR 367–390; range, 350–399).

### Corneal Topography

The median Kmax on Pentacam was 61.4 diopters (IQR 55.2–67.4) preoperatively and 57.2 diopters (IQR 52.5–66.1) at the six-month postoperative visit; *P* = 0.11. The keratometry estimate decreased by a median of 1.1 diopters (IQR 3.2-diopter decrease to 0.2-diopter increase) over the six-month period, with five subjects exhibiting regression (defined as a reduction of Kmax of ≥ 1 diopter), one subject developing progression (defined as an increase of Kmax of ≥ 1 diopter), and the remainder maintaining a relatively stable topography (change in Kmax of < 1 diopter).

### *In Vivo* Confocal Microscopy (IVCM)

**Figure 1:**
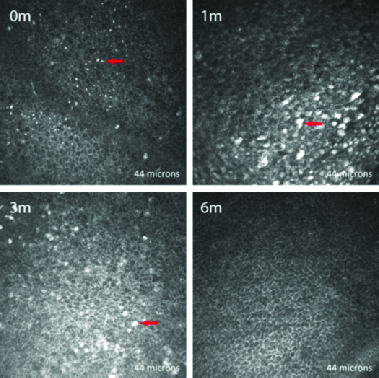
*In* *vivo* confocal microscopic images of the epithelium in a representative patient, taken at the same level at each visit. The epithelium appears normal preoperatively (0 m) which showed minimal disorganization at one month (1 m) and three months (3 m) but normalization at six months (6 m). Microdot cells (arrows) were hyperreflective and showed an increase in size at one month and gradually reverted to original state over time.

The findings noted in the central 8 mm of the keratoconic corneas are as follows:

**Epithelium: **All layers of the corneal epithelium showed normal cell morphology preoperatively in all corneas. Of note, the VKC patients did not show any additional distinct changes in the epithelium before treatment. At the one-month postoperative period, the epithelium was disorganized in all study subjects, with variably irregular cell shape and size compared to the preoperative images. At three months, these changes in epithelial cell architecture had started to resolve, with complete normalization in all corneas by six months [Figure 1]. Three corneas, two of which were from patients with VKC, displayed hyper-reflective globular cells, that is, microdot cells between epithelial cells, preoperatively; all three corneas showed an increase in hyper-reflectivity and size post CXL. In two additional patients with VKC, microdot cells were absent preoperatively but developed in the early post CXL period. In all cases, these hyper-reflective microdot cells decreased in number and size by six months.

**Figure 2:**
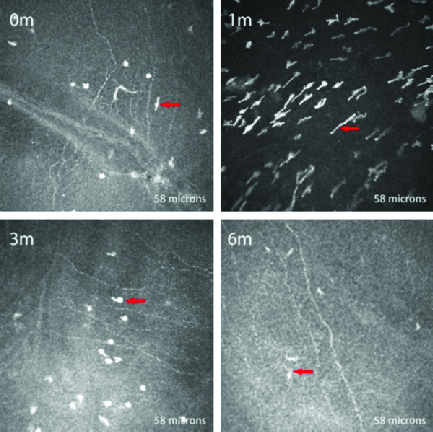
*In vivo* confocal microscopic images of Bowmans membrane in a representative patient, taken at the same level at each visit. The preoperative scan shows tortuous nerve fibers (0 m). Nerves appear fragmented at one month (1 m) and three months (3 m); nerve regeneration is seen at six months (6 m). dendritic cells (arrows) are also seen, which appear hyper-reflective and elongated after crosslinking and revert to the original morphology by six months.

**Figure 3:**
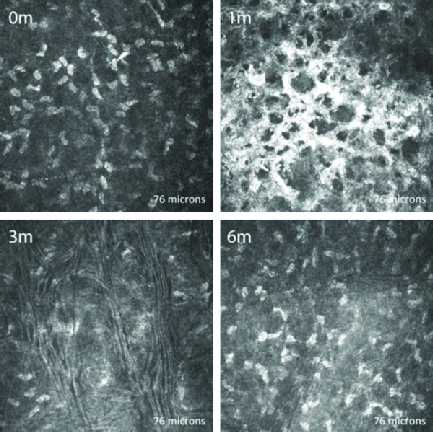
*In vivo* confocal microscopic images of the anterior stroma in a representative patient, taken at the same level at each visit. The scan shows normal keratocytes preoperatively (0 m). Post crosslinking, the anterior stroma showed hyper-reflective cytoplasm and extracellular lacunae in a honeycomb-like appearance with absence of keratocytes at one month (1 m) and gradual keratocyte regeneration at three months (3 m) and six months (6 m).

**Figure 4:**
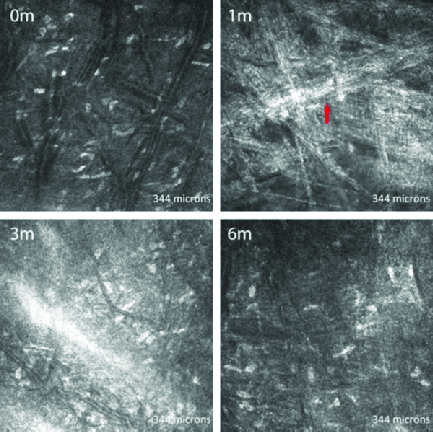
*In vivo* confocal microscopic images of the posterior stroma in a representative patient without posterior stromal apoptosis. Compared with the preoperative scan (0 m), the one-month scan shows new collagen fibers (1 m; arrow), but the keratocyte density is not affected at three months (3 m) neither at six months (6 m) after crosslinking.

**Figure 5:**
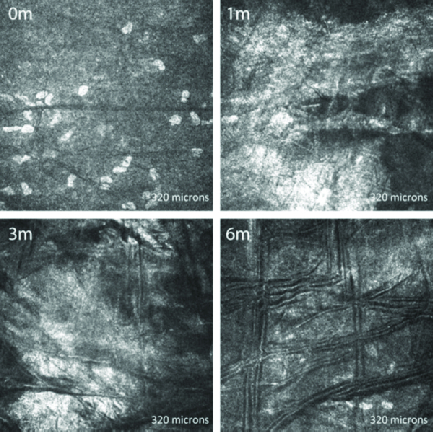
*In vivo* confocal microscopic images of the posterior stroma in a representative patient with posterior stromal apoptosis. Compared with the preoperative scan (0 m), a reduction in the number of keratocytes was observed at one month (1 m), three months (3 m), and six months (6 m).

**Figure 6:**
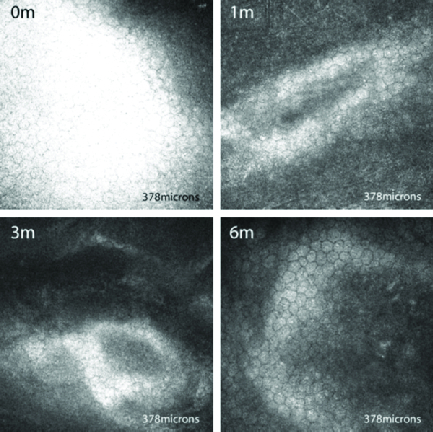
*In vivo* confocal microscopic images of the endothelium of a representative patient, taken at the same level at each visit. The endothelial morphology and density appeared normal preoperatively (0 m) and at one-, three-, and six-month visits (0 m, 3 m, and 6 m, respectively).

### Sub-basal Nerve Plexus

Preoperatively, 10 corneas had a tortuous and branching sub-basal nerve plexus, and 2 showed a beaded appearance as well. At the first postoperative month, the nerve plexus could not be visualized in any of the corneas. By three months, six (60%) subjects exhibited at least some regeneration of the nerves, visualized as small, fragmented nerve fibers with neuritic flocculation. By six months, 8 of the 10 corneas had nearly total regeneration of the subepithelial nerve plexus, except that nerve interconnections were not complete [Figure 2]. In the other two corneas, nerve regeneration was slow and only small, fragmented structures were seen even at six months. Three corneas (30%) showed the presence of dendritic cells preoperatively, two of whom had VKC. In all three corneas, the dendritic cells appeared hyper-reflective and elongated one month after CXL, with a reduction in hyper-reflectivity at three months and absence of hyper-reflectivity at six months, at which time they appeared similar to their preoperative structure.

### Stroma

At one month postoperatively, all corneas showed significant keratocyte apoptosis, with a honeycomb pattern that was more evident in the anterior to mid stroma. The posterior stroma of all corneas displayed dense hyper-reflective needle-shaped bands in a net-like pattern at the one-month visit. Keratocyte apoptosis extended up to the mid stroma in all corneas, and in four patients extended up to the posterior stroma, although the extensive hyper-reflectivity of the stroma made identification of keratocytes difficult. By three months, all subjects showed keratocytes repopulating the stroma, which was more evident in the anterior stroma. The hyper-reflective appearance of the anterior stroma was still evident but the honeycomb appearance had generally diminished. Hyper-reflective bands similar to those seen in the posterior stroma at one month were now seen at the mid stromal level. By six months post CXL, all subjects had a marked reduction in the hyper-reflectivity and complete absence of the honeycomb pattern in the anterior stroma. The stromal keratocyte population had generally increased compared to the previous visits [Figures 3 and 4], though four corneas continued to show deep posterior stromal keratocyte depletion [Figure 5].

### Keratocyte Density

The median anterior stromal keratocyte density was 572 (IQR 497–681) preoperatively, decreased to 266 (IQR 110–385) at the three-month postoperative visit (*P* = 0.005), and then increased to 368 (IQR 243–487) by six months (*P* = 0.04 compared to three months and *P* = 0.007 compared to preoperative) [Table 1]. The median preoperative posterior stromal keratocyte density was 264 (IQR 254–284), which decreased slightly, but not statistically significantly, at the three-month (median 202, IQR 71–281; *P* = 0.17) and six-month visit (median 175, IQR 72–291; *P* = 0.96 compared to the three-month visit and *P* = 0.14 compared to preoperative) [Table 2].

**Table 1:** Quantitative analysis of anterior stromal keratocyte density before and after hypotonic collagen crosslinking.


**Anterior stromal keratocyte density**			
	*Median*	*IQR*	*P-value*
Pre-op	572	497–681	–
Three month	266	110–385	0.005
Six month	368	243–487	0.007
IQR, interquartile range			

**Table 2:** Quantitative analysis of posterior stromal keratocyte density before and after Hypotonic collagen crosslinking


**Posterior stromal keratocyte density**			
	*Median*	*IQR*	*P-value*
Pre-op	264	254–284	–
Three month	202	71–281	0.17
Six month	175	72–291	0.14
IQR, interquartile range			

**Table 3:** Analysis of endothelial cell density before and after hypotonic collagen crosslinking


	*Median ECD (cells/mm ${}^{2}$ )*	*IQR*	*P-value*
Pre-op	2895	2786–2907	–
Post-op	2660	2451–2793	0.005
ECD, endothelial cell density; IQR, interquartile range			

### Endothelium

The endothelial cell morphology was normal in all patients preoperatively and at all subsequent study visits [Figure 6].

### Confocal Findings of Progression 

One subject had progression of keratoconus over the six-month follow-up period, with an increase in Kmax of 6 diopters. This subject was 12 years old, had VKC, and had a family history of keratoconus. The confocal findings of this participant's cornea was most notable for having a very thin Bowman's membrane, breaks in the Bowman's membrane and also being one of the two described above cases that had poor nerve regeneration. Otherwise, confocal findings were similar to the corneas that did not experience progression.

**Peripheral cornea:** An area of inferior peripheral cornea outside the central treatment area was examined as an internal control. The epithelium and stroma of the peripheral cornea was normal in all study participants, with no evidence of treatment effects at any stages of the follow-up.

### Specular Microscopy

Although all subjects had a reduction in endothelial cell density (median reduction 263 cells/mm ${}^{2}$ , IQR 90–342), none showed any signs of endothelial damage clinically (i.e., corneal oedema). The median endothelial cell density as assessed by automated specular microscopy is depicted in Table 3.

##  DISCUSSION

IVCM showed profound morphological changes in corneal cells and structures following hypotonic riboflavin CXL. After the procedure, epithelial cell morphology returned to normal by six months; however, the changes in the subepithelial nerve plexus, stroma, and keratocytes did not. Anterior keratocyte density was markedly reduced after CXL and subsequently increased but did not return to normal by the six-month postoperative visit. Endothelial cell density decreased after CXL, although this reduction was not accompanied by morphological changes and did not seem to be clinically meaningful by six months of follow-up.

Although several studies have reported the IVCM findings after CXL on corneas > 400 μm, very few have documented the changes specifically for thin corneas (i.e., < 400 μm). Those that have assessed thin corneas have studied techniques using isotonic riboflavin.^[19][20,21]^ Our study is novel because it documents the corneal changes post CXL in thin corneas treated with hypotonic riboflavin. While Raiskup^[22]^ and Fahedi^[12]^ have described the efficacy of CXL with hypotonic riboflavin in thin corneas, the corneal structural changes analyzed by IVCM have not yet been reported. Iatrogenic swelling of the cornea with hypotonic riboflavin solution increases the stromal thickness by 25% after 30 min.^[23]^ Hypotonic riboflavin CXL takes advantage of this phenomenon in an attempt to prevent the crosslinking procedure from damaging the endothelium.

The epithelial changes in hypotonic CXL were generally similar to that of isotonic CXL, with transient changes that returned to normal within six months. However, patients with VKC showed distinct structural features including presence of microdot cells, and dendritic cells, which became more pronounced after CXL and gradually reverted to their quiescent state over three to six months post CXL. Previous studies have described these cells in normal individuals and those with VKC,^[24]^ and other inflammatory conditions like post Femtosecond Laser-assisted Keratoplasty^[25]^ and contact lens use,^[26]^ but we are not aware of any reports of their appearance post CXL. Their presence did not seem to correlate with topographic changes post CXL, however, since they are thought to indicate inflammation, they may be worthy of further study in patients with VKC and KCN.

Previous studies of isotonic CXL have reported loss of sub-basal nerves in the early postoperative period, with regeneration as disconnected nerves between the second and third postoperative months, and eventually the appearance of interconnected fibers at 6–12 months.^[15,17,18]^ In contrast to what has been reported for isotonic CXL, sub-basal nerve regeneration after hypotonic CXL was not uniform in our patients. Two of the ten subjects showed very slow nerve regeneration, with a short, fragmented appearance and low density even at six months. It is unclear whether this response represents an expected variation in the healing response, or if this reflects a difference in response to hypotonic riboflavin.

A marked stromal honeycomb pattern suggestive of edema was present one month postoperatively and gradually decreased over the next months until completely absent by six months. This honeycomb pattern was seen more in the mid stroma than in the anterior stroma, possibly because the anterior stroma is more compact compared to the mid stroma. The posterior stroma contained hyper-reflective bands at the one-month post-CXL visit, suggestive of new collagen fibers synthesized by the activated keratocytes. By three months, similar-appearing bands were seen at the anterior mid stromal level. This confocal observation has been suggested to be due to anterior migration of the collagen bands.^[27]^

Some studies of isotonic riboflavin CXL have used changes in keratocyte density to document a demarcation line around 300–320 μm posterior to the epithelium that marks the transition from the treated zone to the untreated zone. Studies of thin corneas have recorded this demarcation line at a slightly deeper location, approximately 320–350 μm posterior to the epithelium.^[19,20]^ It was difficult for us to comment on such a demarcation line since the hyperreflectivity of the stroma precluded an easily visible transition zone.

Although the IVCM analysis of the endothelium did not show any morphological changes at any point postoperatively, the endothelial cell count density was significantly lower at six months than at the preoperative visit. The impact of this finding is not clear. Given the treatment of such thin corneas, it is possible that the crosslinking procedure caused endothelial damage that was not evident morphologically on confocal microscopy. Similar reductions have been observed in some studies of isotonic riboflavin CXL^[28]^ and also in one study of thin corneas^[19]^ but not in any others.^[19,20]^ None of our patients developed corneal edema at any stages of the follow-up, suggesting that the reduction in endothelial cell density was not clinically meaningful over the six months of the study. Nevertheless, this is an important finding that should be studied further.

Many of the findings in the present study were similar to those reported previously following standard or isotonic CXL treatment, including the development and resolution of stromal hyper-reflectivity and stromal honeycomb morphology, as well as the sub-epithelial nerve plexus destruction and regeneration. However, we also noted some differences. The most important difference was the depth of keratocyte apoptosis. Previous studies of isotonic riboflavin CXL,^[13,14][15]^ as well as those specifically performed on thin corneas with CACXL^[19]^ and customized epithelial debridement^[20,21]^ CXL have described structural changes and keratocyte apoptosis limited to anterior to mid stroma. In contrast, we found keratocyte apoptosis extending to mid stroma in six subjects, and to posterior stroma in four subjects assessed by the presence of apoptotic cells in posterior stroma and also by the decreased keratocyte density in posterior stroma in these patients post CXL. We speculate that this could be attributed to fluctuating corneal hydration during the CXL procedure with hypotonic CXL. In addition, we noticed marked dendritic and microdot globular cell activation not previously observed in isotonic riboflavin CXL studies; however, these changes mostly occurred in patients with VKC. It is difficult to determine whether the differences are due to differences in patient populations or surgical technique.

The chief limitation of our study is the small number of subjects included. The efficacy and safety of this technique in the long-term needs to be assessed in subsequent studies with a greater number of patients. Also, four patients in our study had history of vernal disease, which could have confounded our findings. Generalizations are difficult to make given the heterogeneous nature of this cohort.

In conclusion, thin corneas treated with hypotonic CXL generally had same confocal microscopy findings as previous reports of isotonic CXL, with the exception of more posterior keratocyte apoptosis in the hypotonic procedure. This more posterior involvement was accompanied by a significant reduction in endothelial cell density, although not by changes in endothelial cell morphology neither by corneal oedema clinically. Further study of hypotonic CXL is warranted to assess the risk of damage more accurately to the endothelium in patients with corneas < 400 μm undergoing CXL.

##  Financial Support and Sponsorship

None.

##  Conflicts of Interest

The authors have no conflicts of interest to declare.
